# HOW INTERNET USE MODERATES THE EFFECT OF PHYSICAL LIMITATIONS ON SUBJECTIVE WELL-BEING IN OLDER ADULTS

**DOI:** 10.1093/geroni/igad104.2071

**Published:** 2023-12-21

**Authors:** Wan Wen, Shannon Mejia

**Affiliations:** University of Illinois Urbana-Champaign, Urbana, Illinois, United States; University of Illinois Urbana-Champaign, Chanpaign, Illinois, United States

## Abstract

Due to geographic barriers and limitations in mobility, older adults with functional limitations are at greater risk of social isolation and lower well-being. The Internet has the potential to help mitigate the negative effects of functional limitations by connecting older adults to health services and meaningful social networks, and thus contribute to their subjective well-being (SWB). This study examined how Internet use could increase access to health behaviors and close friendships and moderate the impact of physical limitations on older adults’ SWB. Using data from 2008 to 2018 U.S. Health and Retirement Study (HRS) (n = 14,178, 76% white, 55% female), this paper used life satisfaction and emotional indicators (positive and negative affect) as measurements of SWB and adopted multilevel random effect models to conduct empirical analysis. The results showed that older adults with physical limitations experienced significant reductions in life satisfaction and positive affect and increases in negative emotions. However, the Internet mitigated the adverse emotional impacts of physical limitations by facilitating the maintenance of older adults’ social networks and health behaviors. These findings support the development of actions to enhance the well-being of older adults with physical limitations and put them as the primary beneficiaries of information and communication technology.

